# Hybrid Dysfunction Expressed as Elevated Metabolic Rate in Male *Ficedula* Flycatchers

**DOI:** 10.1371/journal.pone.0161547

**Published:** 2016-09-01

**Authors:** S. Eryn McFarlane, Päivi M. Sirkiä, Murielle Ålund, Anna Qvarnström

**Affiliations:** 1 Animal Ecology/ Department of Ecology and Genetics, Norbyvägen 18D, SE-752 36, Uppsala, Sweden; 2 Finnish Museum of Natural History, Zoology Unit, University of Helsinki, Helsinki, Finland; 3 Section of Ecology, Department of Biology, University of Turku, Turku, Finland; University of California Santa Barbara Counseling Services, UNITED STATES

## Abstract

Studies of ecological speciation are often biased towards extrinsic sources of selection against hybrids, resulting from intermediate hybrid morphology, but the knowledge of how genetic incompatibilities accumulate over time under natural conditions is limited. Here we focus on a physiological trait, metabolic rate, which is central to life history strategies and thermoregulation but is also likely to be sensitive to mismatched mitonuclear interactions. We measured the resting metabolic rate of male collared, and pied flycatchers as well as of naturally occurring F1 hybrid males, in a recent hybrid zone. We found that hybrid males had a higher rather than intermediate metabolic rate, which is indicative of hybrid physiological dysfunction. Fitness costs associated with elevated metabolic rate are typically environmentally dependent and exaggerated under harsh conditions. By focusing on male hybrid dysfunction in an eco-physiological trait, our results contribute to the general understanding of how combined extrinsic and intrinsic sources of hybrid dysfunction build up under natural conditions.

## Introduction

Ecological speciation, where barriers to gene flow are the result of divergent adaptation to environmental conditions, is thought to be a common mechanism by which new species form [1–3]. Empirical studies on ecological speciation have generally been focused on young radiations and hence a subset of sources of reproductive isolation, which typically do not include genetic incompatibilities affecting hybrid viability or fertility [1, 2, 4]. Such incompatibilities are likely to evolve slowly and accumulate over time [5, 6], but whether and how ecological divergence leads to a gradual build up of genetic differences that cause combined effects of environmental and physiological mismatches in hybrids remains largely unexplored.

Hybrid dysfunction caused by interacting genes that have diverged in different populations [7, 8] can be the result of random genetic drift, coevolution resulting from genetic conflict or divergent natural and sexual selection [9, 10]. Parallel ecological adaptation resulting from the same selection pressures can also result in genetic incompatibilities if these similar adaptations have evolved due to the fixation of different mutations in different populations [10, 11]. Evidence for Dobzhansky-Muller (DM) interactions causing hybrid lethality or sterility have been demonstrated in, for example, *Drosophila* [12] and *Mimulus* [13] and are expected to be fairly common, although difficult to detect and measure in wild systems.

Although genetic incompatibilities are typically assumed to be between nuclear genomes they may in fact be more common between mitochondrial (mtDNA) and nuclear genomes [14]. A major reason for this assumption is that the mitochondria mutates quickly and has little opportunity for recombination, and thus may be even more susceptible to fix slightly deleterious mutations that could be incompatible on a different nuclear background [14, 15]. Mitonuclear interactions may be underlying ecological adaptations as well because of the mitochondria’s role in oxidative phosphoralation (OXPHOS) forming ATP [14, 16] and may therefore play an important role in the speciation process [16], especially in relation to climate adaptation. Climate adaptation is however surprisingly rarely studied in the context of ecological speciation, and this is especially true in relation to the potential build up of genetic incompatibilities (reviewed in [17]).

Resting metabolic rate is an ecologically influenced physiological trait which can be defined as the baseline energy needed to run an individual’s organs, and hence influences its life history strategy [18]. Metabolic rate affects thermoregulation [19] and, in hybrids, is expected to be different from the parental species if there are DM interactions between the mitochondria and nuclear genomes contributing to OXPHOS. This has been found in seed beetles, where metabolic rate is affected by an interaction between the mitochondria, the nuclear background and the environment [20]. Similar results have also been found in studies on *Drosophila*, where hybrids between *D*. *melanogaster* and *simulans* had a higher metabolic rate than the parental species at warmer temperatures, which could be mapped to a mitonuclear interaction [21]. In birds, breeding experiments of stonechats revealed differences in metabolic rate among individuals with the same nuclear but different mitochondrial genomes [22]. These laboratory studies are often based on different isolines where mitochondria are “placed” into an entirely different genomic background (i.e. the mitochondrial genome comes from one isoline and the nuclear genome from another) and can therefore not directly be applied to expectations concerning hybrids and early backcrosses. Evidence for disrupted metabolic rate in naturally occurring hybrids is scarce and it therefore remains an open question whether this is a common physiological defect in hybrids.

Natural hybrid zones provide the opportunity to both identify phenotypic traits that are related to ecological speciation and possible links to hybrid dysfunction [23]. Collared (*Ficedula albicollis*) and pied (*F*. *hypoleuca*) flycatchers are a suitable system in which to study the effects of climate adaptation on speciation. These two species of closely related passerines diverged less than one million years ago [24], and have both allopatric and sympatric populations, including recent hybrid zones on the Swedish islands of Öland and Gotland in the Baltic [25]. Generally, collared flycatchers have a more southern breeding distribution, while pied flycatchers are more northerly distributed [17, 25]. Collared and pied flycatchers differ in life history strategies, where collared flycatchers trade hardiness for competitive dominance [26, 27]. Differences in climate adaptation between these two species, as reflected by differences in breeding ranges and resource allocation strategies, may be expected to have resulted in divergence in RMR and the underlying genes. Hybrids could then either have an intermediate metabolic rate with mainly ecological implications or show signs of a more severe mismatch. Laboratory studies suggest that mismatched mitonuclear lines rather have a deviating (i.e. either higher or lower metabolic rate when compared to the parental species) than an intermediate RMR.

## Methods

We monitor collared and pied flycatchers during their breeding season on the Swedish island of Öland, where they co-occur [25]. Both species are obligate cavity nesters [28], and readily nest in nestboxes we have placed in woodlands on the island. Additionally, some birds were caught as part of a constant effort mist netting at the Ottenby Bird Observatory at the southern tip of Öland. All experimental procedures on flycatchers reported here were approved by the Linköping animal care committee, Kalmar länstyrelse gave us permission and all local land owners gave their permission to conduct the study on their land. Approximately 4% of breeding flycatchers in this population are hybrids [29]. All birds caught were ring marked with a unique alphanumeric metal band, weighed, blood sampled, and measured for a variety of morphological measurements, including tarsus length, wing length and plumage characteristics, as part of the long term monitoring program. Species were assigned phenotypically by plumage characteristics while the bird was in hand. To assign hybrids as having either collared maternity or pied maternity, we used one mitochondrial marker that has been found to be fixed in either species [30].

We measured resting metabolic rate of males during the breeding seasons in 2013–2015 on Öland. Males were caught using either mist nets and playback, or were trapped in nest boxes, and were caught haphazardly throughout the entire breeding season, including while they were migrating, advertising for females, and feeding nestlings. Males were either caught in the evening, or caught in the morning while defending their territories and kept in cages with ad lib food until evening. We preferentially captured males to bring to the laboratory, as males without females are likely to abandon their nestlings, but single females will continue to feed the brood (S.E. McFarlane, pers obs). RMR of all males was measured at night, either between 8pm to 12am or 12am to 4am. Overnight, while we were measuring RMR, males were kept in a climate-controlled cabinet set at 28°C to ensure that they were in their thermoneutral zone [31]. We used a FMS respirometer, RM-8 multiplexer, PP-2H field pump and a FlowBar-8 (Sable Systems, Henderson, NV, USA). With this arrangement we were able to measure up to seven birds at a time, with a reference chamber. The multiplexer cycled through chamber measurements, where birds were measured 6 times for five minutes each, where each cycle took 40 minutes and the males were in the chambers for four hours in total. We automatically discarded the first measurement of the six, as we assumed that the bird was not yet settled. From the other five measurements, we have used here the sample with the lowest standard deviation among each sampling second. We assume that this low variation among samples represented a calm or sleeping bird. The flow rate for each chamber was set to approximately 400ml/minute, and recorded once per second. We used equations 9.3 and 9.4 in Lighton [32] to calculate VO_2_, from O_2_, CO_2_ and water vapor pressure. To estimate mass standardized metabolic rate, we divided each VO_2_ by the mass of the bird immediately before it was measured. We present mass standardized metabolic rate as milliliters per minute per gram. In the results, we report mean estimates +/- standard deviations.

To investigate whether there is a species effect on metabolic rate, we used a mixed effects model using “lme4” in R v 3.0.2, where metabolic rate was the dependent variable, species was the independent variable, and year was included as a random effect [33, 34]. We report here results from an ANOVA of this mixed effects model, assuming the Satterthwaite approximation for degrees of freedom, which we implemented using “lmerTest” [35]. We did post hoc comparisons between species to determine the drivers of the species difference using Tukey contrasts for mixed effects models from the ‘multcomp’ package [36].

To determine if hybrids from each cross type (i.e. different maternal species) differed in metabolic rate, we did a Student’s t test in R [34].

## Results

In total, we measured the metabolic rate of 94 male *Ficedula* flycatchers (39 collared flycatchers, 43 pied flycatchers and 12 hybrids), between 2013 and 2015. The mean resting metabolic rate was 3.49+/- 1.7 ml of oxygen consumed min^-1^, and the mean mass standardized metabolic rate was 0.271+/-0.13, where the mean mass was 12.88+/-0.99 grams. We found that both collared flycatchers and hybrids were heavier than pied flycatchers (PF-CF est = -1.01+/-0.18, z = -5.49, p<0.0001, PF-HY est = -1.033+/-0.29 z = -3.62, p = 0.0008), although collareds and hybrid flycatcher males were overlapping in size (est = -0.023+/-0.28, z = 0.084, p = 0.996). We also found that hybrids did not differ significantly in tarsus length from either pure species (CF-HY est = 0.204+/-0.23, z = 0.873, p = 0.65, PF-HY est = -0.187+/-0.23, z = -0.805, p = 0.69) but that collared flycatchers had significantly longer tarsi than pied flycatchers, suggesting that hybrids are intermediate to the parental species (est = 0.39+/-0.15, z = 2.56, p = 0.027). Here we have presented both absolute metabolic rate and mass standardized metabolic rate, although mass was not a significant predictor of metabolic rate (F_1, 91.5_ = 0.69, p = 0.408). We found no effect of tarsus on metabolic rate (F_1, 85.9_ = 0.438, p = 0.510), and so report mass standardized metabolic rate as a control for size.

**Fig 1:**
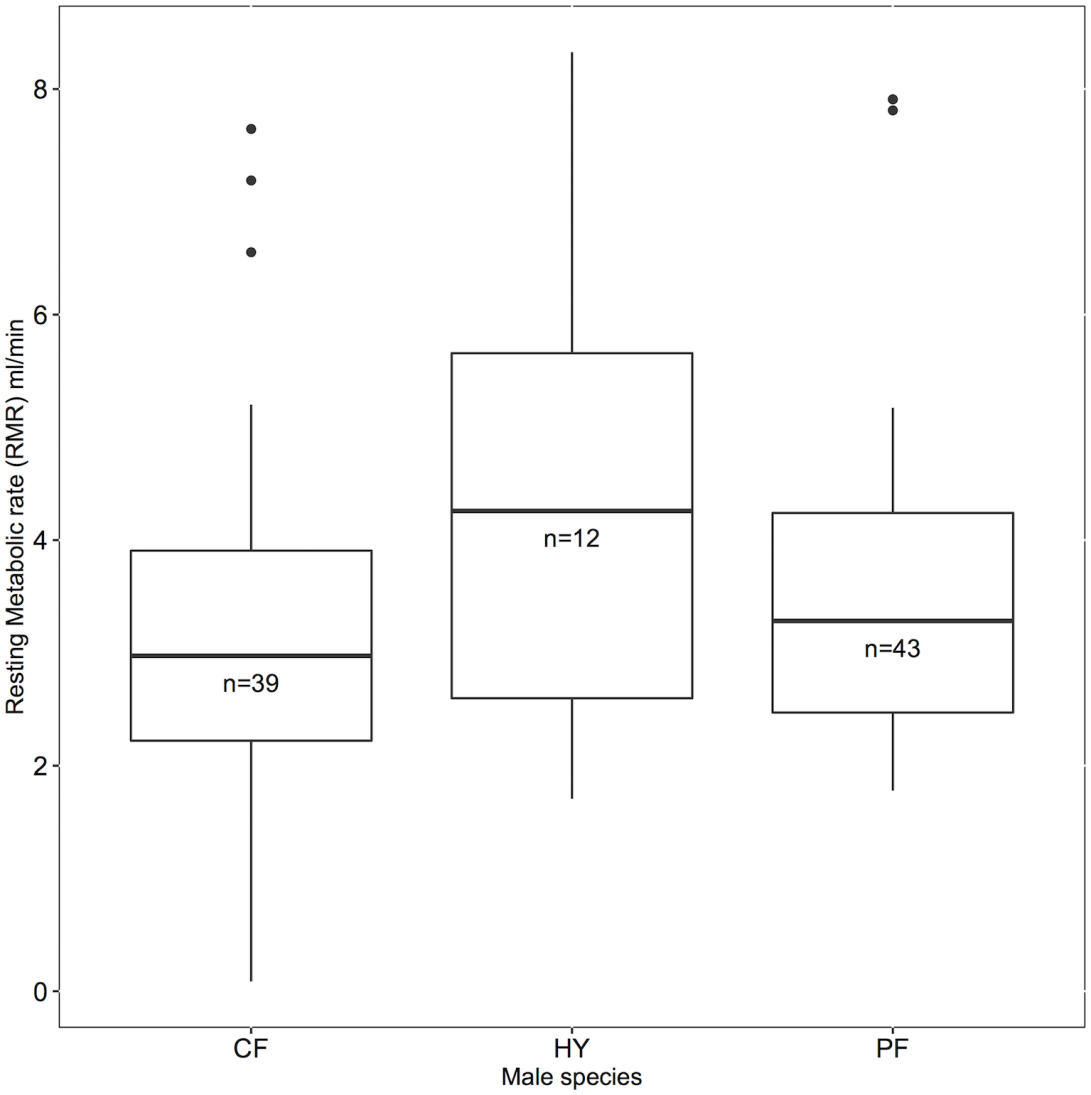
A comparison of whole organism resting metabolic rate (ml/minute) among collared (CF, n = 39), pied (PF, n = 43) and hybrid (HY, n = 12) male flycatchers breeding in 2013, 2014, and 2015 on Öland. We found that hybrid males tended to have higher metabolic than either parental species.

We found a marginally significant species effect on metabolic rate (F_2,90.9_ = 2.66, p = 0.076), which was driven by the differences between hybrid and collared flycatcher males (est = 1.22+/-0.53, z = 2.30, p = 0.055), and somewhat between hybrid and pied males (est = 0.872+/-0.53, z = 1.65, p = 0.22). This is consistent with our prediction that hybrids have a different metabolic rate because of mismatched mito-nuclear interactions (Fig 1). Additionally, collared males and pied males were not discernibly different from each other (est = -0.348 +/-0.36, z = -0.98, p = 0.58).

The difference in size between collared and pied flycatcher males was not causing the observed differences in metabolic rate, as metabolic rate standardized by mass was also marginally different in hybrids compared to collared flycatcher males (HY-CF est = 0.090+/-0.04, z = 2.13, p = 0.082), although it was not different between hybrid males and pied flycatcher males (PF-HY est = -0.041+/-0.043, z = -9.73, p = 0.59), and the difference between collared and pied males was not significantly different from zero (est = -0.05+/-0.03, z = -1.78, p = 0.17).

**Fig 2:**
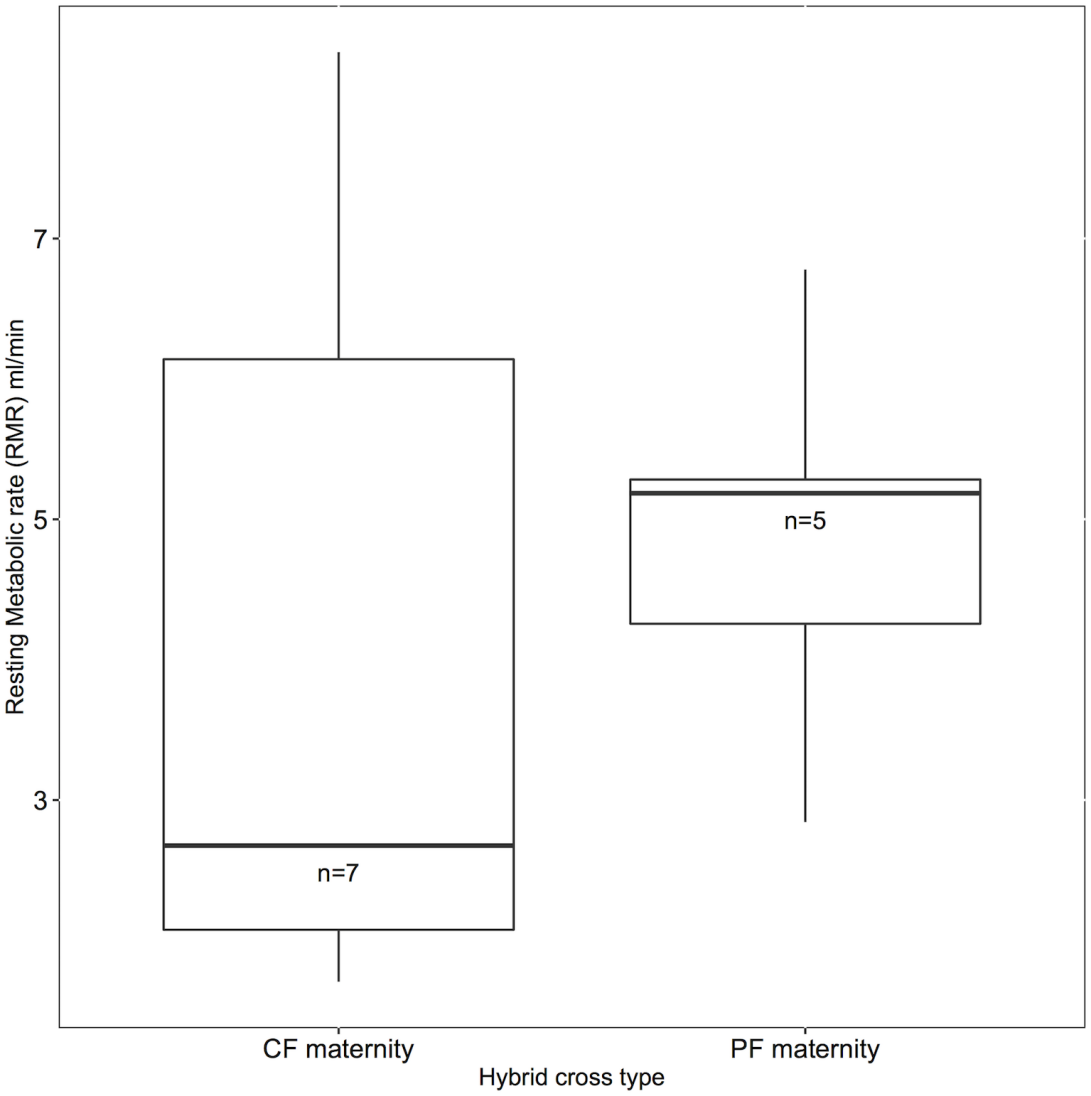
We tested whether male hybrids with collared flycatcher mtDNA (CF mtDNA, n = 7) or pied flycatcher mtDNA (PF mtDNA, n = 5) had different whole animal metabolic rates (ml/minute). We found no significant evidence of a difference between cross types.

We did not find an asymmetric relationship between cross types, as hybrids from pied flycatcher mothers and from collared flycatcher mothers did not differ significantly in metabolic rate (t = -0.559, df = 9.27, p = 0.589; Fig 2).

## Discussion

We report elevated metabolic rate of male F1 hybrids resulting from both types of pairing combinations between pied and collared flycatchers. An elevated metabolic rate means that self-maintenance is more costly for these hybrids and hence likely reflects hybrid physiological dysfunction.

If everything else is held equal, a higher resting metabolic rate is costly because of the need to allocate more energy for maintenance [18]. Individuals with lower metabolic rates can survive longer [37], and grow faster [38], likely because, instead of allocating energy towards maintenance, individuals with low metabolic rate can allocate their energy to other life history traits (i.e. the compensation hypothesis;[39]). However, when individuals are challenged, such as with increased workload during reproduction, they may respond by raising their resting metabolic rate in order to be able to increase their daily energy expenditure (DEE;[40]). For example, over the course of lactation, laboratory mice were found to increase their RMR to keep up with the associated increased energetic demands [41]. In addition, experimental manipulations of reproductive effort by brood size enlargement have been shown to increase RMR of the attending parents [42, 43]. Thus, a higher RMR may reflect a plastic response to a needed, higher DEE. Hybrid flycatchers have been shown to have lower pairing success [29], and to be more likely to raise extra pair offspring than either collared or pied flycatcher males [44], and thus might need to have a higher DEE to attract females, and then to guard them. The observed substantially higher mean metabolic rate of male hybrid flycatchers compared to males belonging to either of the two parental species (Fig 1) may therefore either represent a difference in energy allocation strategy such that hybrids invest relatively more in reproduction (i.e. to compensate for other sources of reproductive dysfunction), or hybrid physiological dysfunction. Future work could disentangle these two possible explanations by examining if and how variation in RMR at different breeding stages (i.e. advertising for females vs. feeding nestlings) affects relative performance of hybrid males.

An association between higher metabolic rate and a higher energy allocation towards reproduction has been found in interspecific studies of mammals. Higher sperm production was linked to species with high mass standardized metabolic rates, suggesting that highly efficient physiologies were needed for high sperm production [45, 46]. However, male hybrid flycatchers have been shown to have severely reduced fertility, in particular, they have fewer, malformed sperm when compared to pure species males [44]. We therefore find it most likely that the elevated metabolic rate that we report here is a sign of hybrid dysfunction in the flycatchers. Malformed sperm is moreover *per se* consistent with the hypothesis of mismatched mitonuclear interactions because sperm production is also affected by OXPHOS [47, 48]. Thus, it is expected that individuals with incompatible mito-nuclear genomes have both poor sperm production and abnormal metabolic rates. We therefore find it most likely that the elevated RMR in hybrid flycatcher reflects physiological dysfunction.

The use of naturally occurring hybrids to test the effects of mitonuclear interactions, especially based on known F1 hybrids, is rare, likely because of the difficulty of catching known hybrids on which to measure physiological traits. A higher mass-standardized RMR was reported in black capped—Carolina chickadee hybrids as compared to the two parental species [49]. However, black capped—Carolina chickadee hybrids did not have a higher whole organism RMR while flycatcher hybrids have both a higher mass-standardized and a higher whole organism metabolic rate, which, all else being equal, is costly [18], and likely represents a symptom of hybrid dysfunction. Further, the flycatcher hybrids we measured were F1 hybrids, as F2 hybrids or backcrosses are exceptionally rare or absent in this population [50], leading to clear instances with mismatched mito-nuclear genomes. This can be compared to the chickadee system, where backcross hybrids in addition to F1 hybrids were measured [49], which may make predictions about mitonuclear interactions more difficult to test, as some backcrosses will not be mismatched (i.e. those individuals without mismatched mitochondrial and nuclear genomes). Italian sparrows, a hybrid species between house sparrows and Spanish sparrows, have been shown to have reproductive barriers with the parental species that are coded on the sex chromosomes and mitochondria, suggesting mitonuclear discordance, although the phenotype involved remains unknown [51]. While much more work is needed, it is possible that avian hybrids, as well as hybrids in other taxa, are regularly affected by mitonuclear discordance.

We did not find strong evidence of an asymmetry between hybrids with either collared or pied mtDNA, but our low sample size (due to the rareness of natural hybrids) makes it premature to conclude that the effects are symmetric. Asymmetry between the specific crosses is expected because of the sex specific genetic contributions (“Darwin’s Corollary”; [52]), i.e. mitochondria are contributed only from the mother, and for this reason F1 hybrids with a collared mother have different genetic material than F1 hybrids that had a pied mother. For example, *Nasonia* wasps have asymmetrical F2 hybrid mortality, which was directly attributed to differences in mitochondrial peptides involved in OXPHOS [53], and [54] found that *Centrarchidae* fishes with faster mitochondrial evolution were worse maternal parents in reciprocal hybrid crosses. Asymmetries may be more pronounced in backcrosses that have the mitochondria of one species and the nuclear genome of the other, compared to F1 hybrids that have both nuclear genomes but only one species mitochondria. In the case of F1 hybrids, dysfunction may be partial, but not complete as would be expected in the above-mentioned subset of F2 or backcross hybrids.

A stressful climate may lead to a higher expression of hybrid dysfunction with mismatched mitochondria and nuclear genomes. For example, mitonuclear lines of seed beetle hybrids (i.e. those with mismatched mitochondrial and nuclear genomes) did not have a different metabolic rate at a normal temperature; the effect was only seen at a higher, stressful temperature [20]. A similar pattern was found in *Drosophila* mitonuclear lines [21]. There is thus the potential for a genotype by genotype by environment interaction affecting metabolic rate. Collared flycatchers have only recently colonized Öland [25], this is the northern extreme of their range. For this reason, the climate on Öland could be an extreme climate, for collared flycatchers and their hybrids. Future research could examine whether the same pattern of hybrids with increased metabolic rate happens in the older *Ficedula* hybrid zone in Central Europe, where the birds may experience a less stressful climate.

Collared flycatchers have been identified as a sensitive species to current climate warming [55], facilitating their move into Northern Europe. As climate change intensifies, more species will have range shifts into newly available habitat [56], face new environmental selection pressures and potentially come into secondary contact and hybridize with closely related species [57]. This hybridization can have either positive [58] or negative [59] impacts on population survival. Recent instances of hybridization between northern and southern flying squirrels have occurred after rapid northern range expansion by the southern species, which was strongly correlated with warmer winters [60]. Metabolic rate is tightly linked to climate adaptation (e.g. [19]), and mismatched mitonuclear interactions achieved through experimental crossings have found that effects on metabolic rate are more pronounced under extreme thermal environments [20, 21]. By increasing our understanding of mitonuclear interactions and metabolic rates of hybrids in naturally forming hybrid zones, we can begin to make predictions about the outcomes of climate change on speciation events through combined effects of range shifts and thermal adaptation.
