# Correction: Hybrid Dysfunction Expressed as Elevated Metabolic Rate in Male *Ficedula* Flycatchers

**DOI:** 10.1371/journal.pone.0181288

**Published:** 2017-07-20

**Authors:** S. Eryn McFarlane, Päivi M. Sirkiä, Murielle Ålund, Anna Qvarnström

There are errors in the second sentence of the first paragraph of the Results. The correct sentence is: The mean resting metabolic rate was 1.20±0.5 ml of oxygen consumed min^-1^, and the mean mass standardized metabolic rate was 0.093±0.03, where the mean mass was 12.88+/-0.99 grams.

There are errors in the final two sentences of the first paragraph of the Results. The correct sentences are: Here we have presented both absolute metabolic rate and mass standardized metabolic rate, although mass was not a highly significant predictor of metabolic rate (F_1, 91_ = 3.40, p = 0.068). We found no effect of tarsus on metabolic rate (F_1, 89_ = 0.036, p = 0.849), and so report mass standardized metabolic rate as a control for size.

There are errors in the first sentence of the second paragraph of the Results. The correct sentence is: We found a significant species effect on metabolic rate (F_2, 91_ =, 5.72, p = 0.0046) which was driven by the differences between hybrid and pied males (est = 0.486± 0.15, z = 3.16, p = 0.0043), and somewhat between hybrid and collared males (est = 0.262± 0.16, z = -1.68, p = 0.207).

There are errors in the third sentence of the second paragraph of the Results. The correct sentence is: Additionally, collared males and pied males were not discernibly different from each other (est = 0.225± 0.10, z = 2.155, p = 0.077).

There are errors in the penultimate paragraph of the Results. The correct paragraph is: The difference in size between collared and pied flycatcher males was not causing the observed differences in metabolic rate, as metabolic rate standardized by mass was also different in hybrids compared to pied flycatcher males (HY-PF est = 0.030±0.01, z = 2.30, p = 0.05), although it was not different between hybrid males and collared flycatcher males (CF-HY est = -0.019±0.01, z = -1.49, p = 0.289), and the difference between pied and collared males was not significantly different from zero (est = -0.010 ± 0.01, z = -1.21, p = 0.438).

There are errors in the final paragraph of the Results. The correct paragraph is: We did not find an asymmetric relationship between cross types, as hybrids from pied flycatcher mothers and from collared flycatcher mothers did not differ significantly in metabolic rate (t = 0.0035, df = 9.99, p = 0.997, Fig 2).

Figs 1 and 2 are incorrect. The authors have provided corrected versions here.

**Fig 1:**
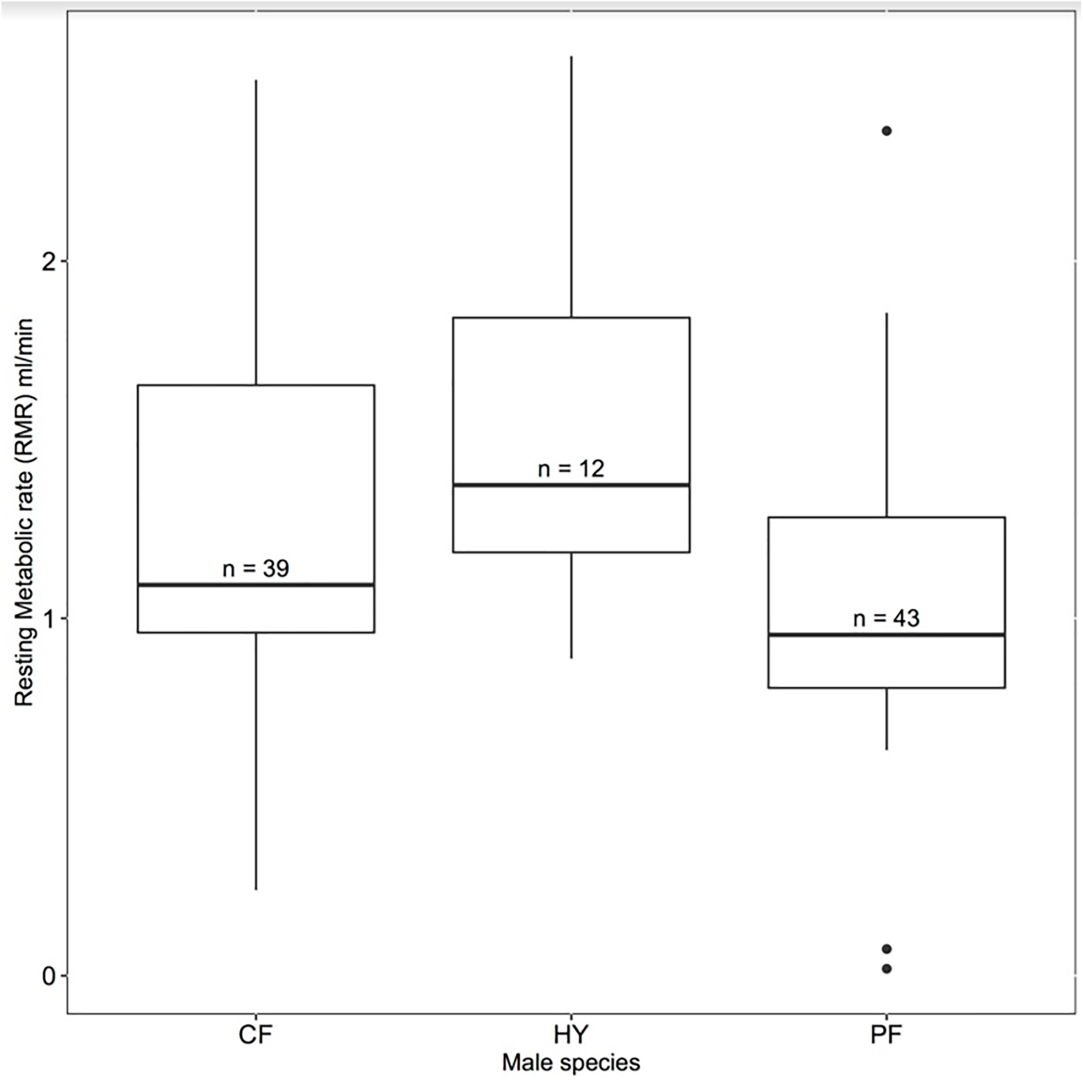
A comparison of whole organism resting metabolic rate (ml/minute) among collared (CF, n = 39), pied (PF, n = 43) and hybrid (HY, n = 12) male flycatchers breeding in 2013, 2014, and 2015 on Öland. We found that hybrid males tended to have higher metabolic than either parental species.

**Fig 2:**
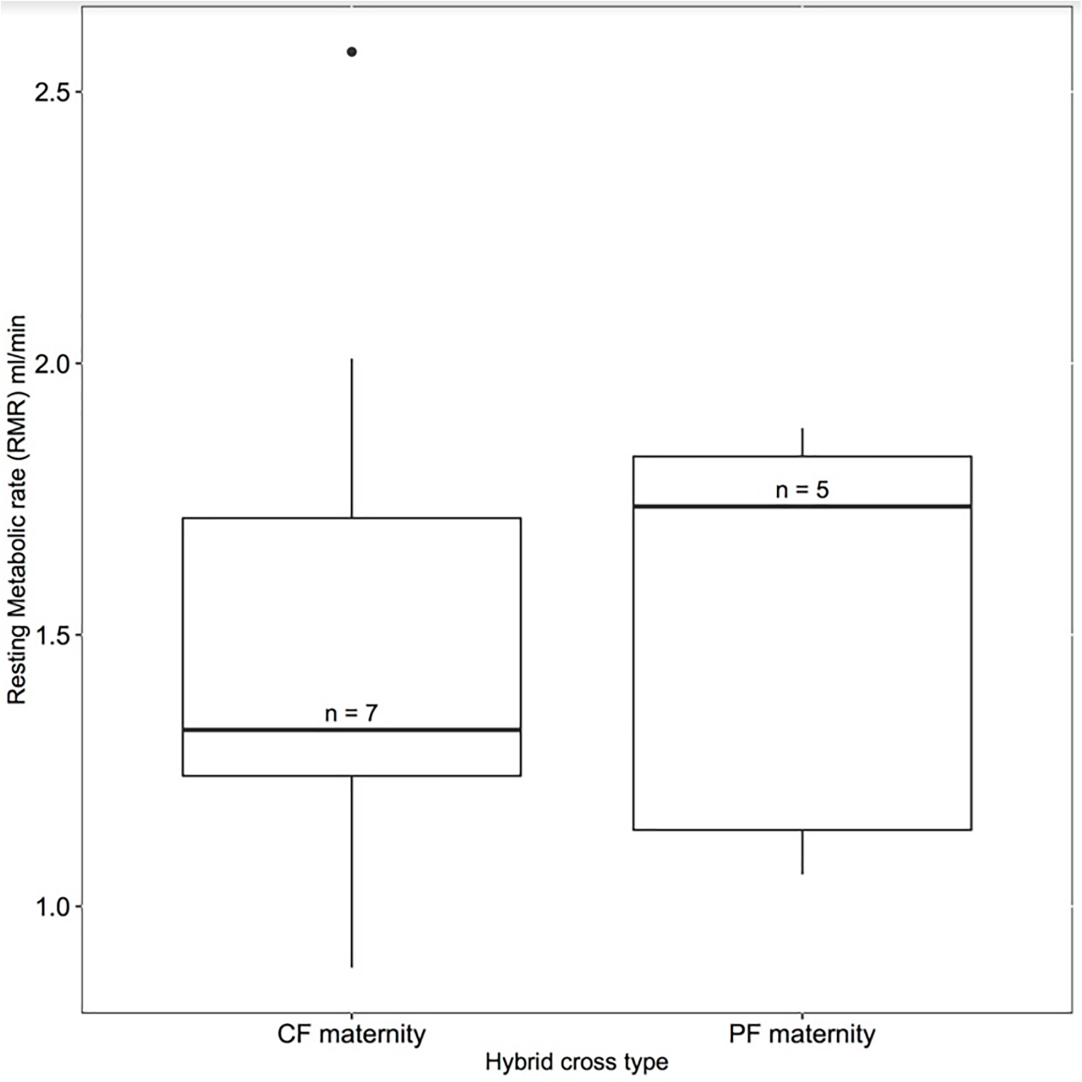
We tested whether male hybrids with collared flycatcher mtDNA (CF mtDNA, n = 7) or pied flycatcher mtDNA (PF mtDNA, n = 5) had different whole animal metabolic rates (ml/minute). We found no significant evidence of a difference between cross types.
